# Colonocyte keratin 7 is expressed de novo in inflammatory bowel diseases and associated with pathological changes and drug-resistance

**DOI:** 10.1038/s41598-022-26603-2

**Published:** 2022-12-23

**Authors:** Lauri Polari, Mervi Tenhami, Santeri Anttila, Terhi Helenius, Harry Kujari, Markku Kallajoki, Markku Voutilainen, Diana M. Toivola

**Affiliations:** 1 Cell Biology, Biosciences, Faculty of Science and Engineering, Åbo Akademi University, BioCity, Tykistökatu 6A, 20520 Turku, Finland; 2 InFLAMES Research Flagship Center, Åbo Akademi University, BioCity, Tykistökatu 6A, 20520 Turku, Finland; 3 Department of Clinical Medicine, University of Turku, Turku, Finland; 4 Division of Digestive Surgery, Turku University Hospital, Turku, Finland; 5 Institute of Biomedicine, University of Turku, Turku, Finland; 6 Laboratory Division, Turku University Hospital, Turku, Finland

**Keywords:** Diagnostic markers, Inflammatory bowel disease, Colon, Intermediate filaments, Immunochemistry

## Abstract

The clinical course of IBD, characterized by relapses and remissions, is difficult to predict. Initial diagnosis can be challenging, and novel disease markers are needed. Keratin 7 (K7) is a cytoskeletal intermediate filament protein not expressed in the colonic epithelium but has been reported in IBD-associated colorectal tumors. Our aim was to analyze whether K7 is expressed in chronic colonic inflammatory diseases and evaluate its potential as a novel biomarker. K7 was analyzed in two patient cohorts using immunohistochemistry-stained colon samples and single-cell quantitative digital pathology methods. K7 was correlated to pathological changes and clinical patient characteristics. Our data shows that K7 is expressed de novo in the colonic epithelium of ulcerative colitis and Crohn’s disease IBD patients, but not in collagenous or lymphocytic colitis. K7 mRNA expression was significantly increased in colons of IBD patients compared to controls when assessed in publicly available datasets. While K7 increased in areas with inflammatory activity, it was not expressed in specific crypt compartments and did not correlate with neutrophils or stool calprotectin. K7 was increased in areas proximal to pathological alterations and was most pronounced in drug-resistant ulcerative colitis. In conclusion, colonic epithelial K7 is neo-expressed selectively in IBD patients and could be investigated for its potential as a disease biomarker.

## Introduction

Keratin 7 (K7) is a type II intermediate filament protein and part of the cellular cytoskeleton primarily in several glandular and ductal epithelia^1^. Apart from serving a structural functions in these cells, the molecular roles of K7 and disease-expression patterns are not well known compared to other simple epithelial keratins^2^. K7-deficient mice have no major reported phenotype, except increased proliferation in the urothelium^3^. While K7 is expressed in the crypts of the mouse colon, K7 is not expressed in the normal human colonic epithelium in which K8, K18, K19, and K20 are major intermediate filaments^1–4^. Likewise, K7 is rarely found in sporadic colorectal adenocarcinomas^5^. However, an exception is ulcerative colitis (UC)-linked adenocarcinomas of which 45–70% of the cases were reported K7 positive^6,7^. This may indicate that K7 expression could be already induced during UC. A similar phenomenon was recently suggested in skin as keratinocyte K17 was upregulated in inflammation preceding tumorigenesis^8^.

UC, along with Crohn’s disease (CD), are major subtypes of inflammatory bowel diseases (IBD). UC affects the colorectal mucosa while CD is a transmural disease that may affect the whole gastrointestinal tract and sometimes other organs^9^. The clinical course of IBD is characterized by relapses and spontaneous or drug-induced remissions. Longstanding IBD is a risk factor for colorectal cancer (CRC), which is not always preceded by a low to high dysplasia sequence typical for sporadic CRC^10,11^. Microscopic colitis is another chronic inflammatory diseases with typical manifestations in the intestinal mucosa. On the contrary to IBD, microscopic colitis, does not promote CRC risk^12^. Microscopic colitis detection requires colonoscopy-obtained biopsies to identify the structural microscopic changes in the colonic epithelium. Microscopic colitis comprises two distinct diseases, lymphocytic colitis (LC) and collagenous colitis (CC), with annual incidences of 5.76/100,000 for LC and 5.48/100,000 for CC in Western countries^13^.

The diagnosis of chronic colon inflammatory conditions is based on symptoms, colonoscopy, pathological findings from biopsies and elevated levels of stool calprotectin. Nevertheless, due to heterogeneity especially in IBD, distinguishing between disease subtypes using current diagnostics methods is challenging, and findings do not fully indicate the prediction of the disease^14,15^. Calprotectin is produced by neutrophils and thus is an acute marker of inflammatory activity, and less related to pathological changes in the epithelium. Currently, there are no good molecular marker to indicate IBD-associated disruptions of epithelial integrity^16^, such as erosion, edema, crypt damages^17^. Among the homeostasis maintaining epithelial cell components, the intermediate filament keratins are major cytoskeletal proteins, and their expression and post-translational modifications are altered in colonic stress conditions^18,19^. As biomarkers, keratins are easy to detect due to their high cellular concentrations and recognizable cytoplasmic expression patterns, and their prognostic value is already recognized and utilized in cancer research and diagnostics^20^. In an attempt to characterize the factors modulating K7 and to evaluate its potential biomarker value for colon diagnostics, we investigate colonic K7 expression in IBD and microscopic colitis patient samples. To our knowledge, this is the first time when K7 expression changes are digitally quantified and compared in four distinct inflammatory non-cancerous colon diseases, and correlations analyzed with clinical characteristics including patient sex, BMI, age, tissue pathology, immune cell subsets present and response to medication.

## Results

### K7 is expressed in the colonic epithelium in UC and CD but not in microscopic colitis

**Figure 1:**
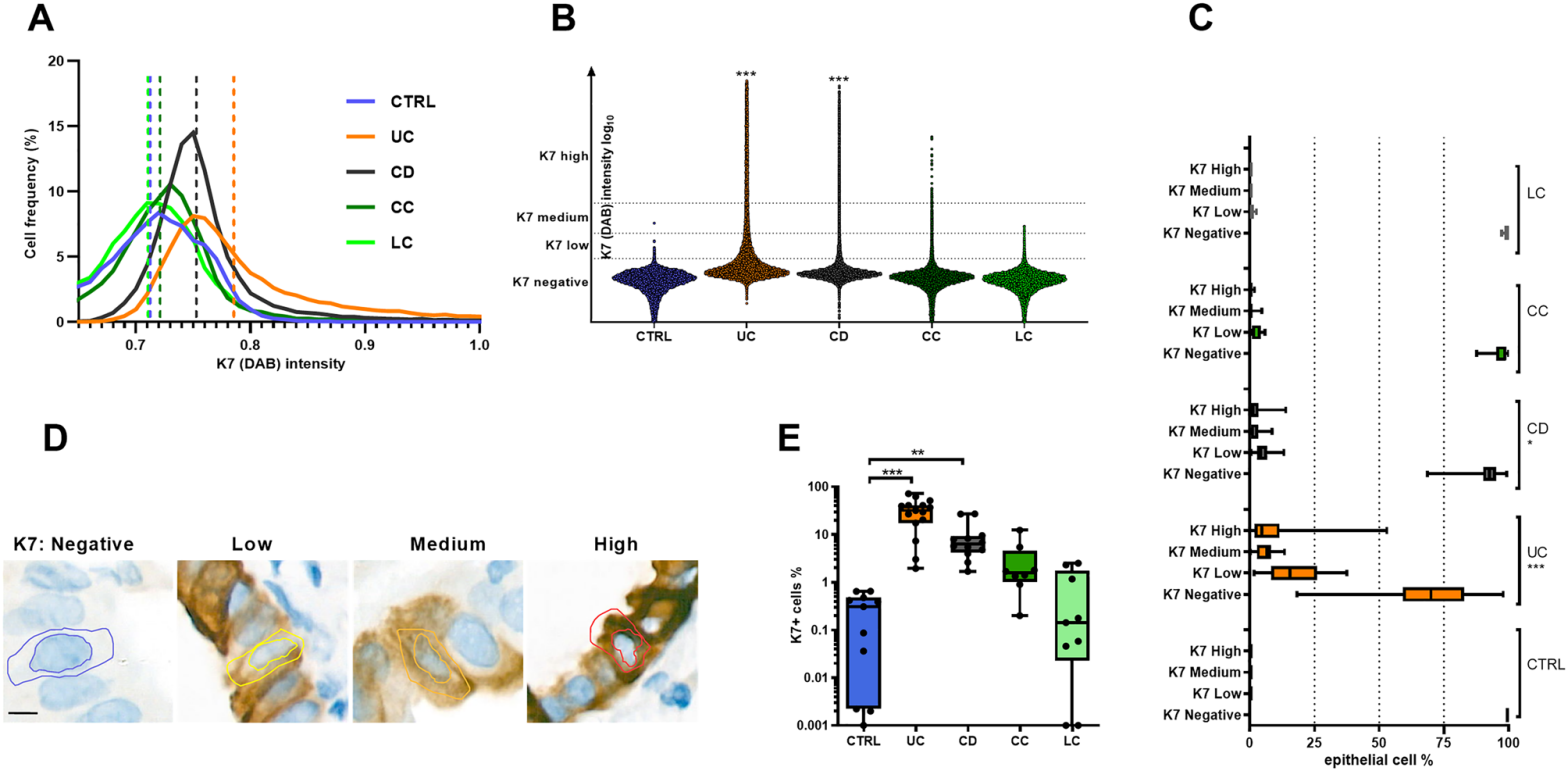
K7 is expressed in UC and CD but not CC and LC. (**A**) Both the frequency of K7 positive cells, shown in histogram with median value pointed out with slashed lines and (**B**) cell populations from annotated areas, presented according to K7 intensity, show that both min, max and median K7 intensities are increased in CD and UC compared to controls. (**C**) Cells are further stratified according to the K7 intensity into four groups (vertical lines in **B**): K7 negative, low, medium, and high K7-expressing cells by digital analysis of K7 stained patient samples, are shown. Medium- and high expressing K7-cells were increased in CD and UC while K7 negativity was decreased. (**D**) Representative examples of K7-stratified cells, where the inner marked area shows the nucleus and the outer the algorithm-predicted cytoplasm. (**E**) The difference in the percentage of K7 positive (K7+) cells between diseases, where one dot represents one patient, is shown. Values in C and E refer to the percentage of epithelial cells in respective group. Boxes extend from 25 to 75th percent, line inside shows median values and whiskers min and max values. Samples presented here belong to cohort A. Scale bar = 5 μm. Statistical significance is based on Dunn’s multiple comparison test. *P < 0.05; **P < 0.01; ***P < 0.001.

**Figure 2:**
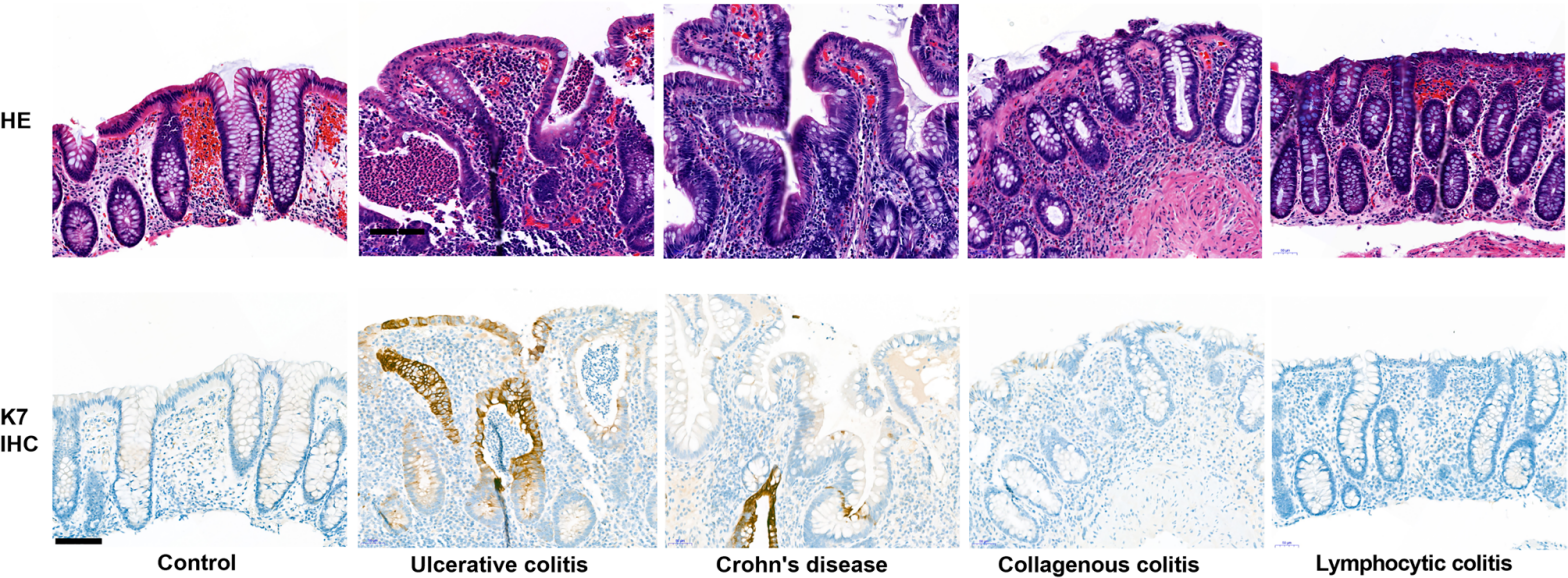
Typical K7 expression patterns in IBD and microscopic colitis. Images show for controls and the disease (ulcerative colitis, Crohn’s disease, collagenous colitis, lymphocytic colitis) representative K7 epithelial expressions (K7 IHC DAB brown color) and H&E staining from the same areas.

The number of K7 expressing epithelial cells in colon was higher in IBD biobank cohort A, witnessed by increased K7 median cellular intensity (Fig. 1A,B). When epithelial cells were stratified into negative, low, medium, and high K7-expressing cells (Fig. 1B), K7 high- and medium cells were relatively common in UC and CD samples but they were not present in controls (Fig. 1C). The classification system used (Fig. 1C) is illustrated by representative examples of individual cells (Fig. 1D). A comparison according to K7 negativity/positivity of cells showed that every CD and UC sample had more K7 positive cells (K7+) than any control sample (Figs. 1E, 2). LC samples had similar negligible K7 levels as controls, while some of the CC samples showed a few focal K7 expressing areas, failing to reach statistical significance. Nevertheless, a limitation in our study is that the number of CC patients was lower compared to those of UC, CD and LC. Thus, our findings for CC need to be confirmed in a larger cohort. Taken together, K7 expression is increased in all studied cases of UC and CD in cohort A.

### K7 upregulation in IBD is detectable at mRNA level

**Figure 3:**
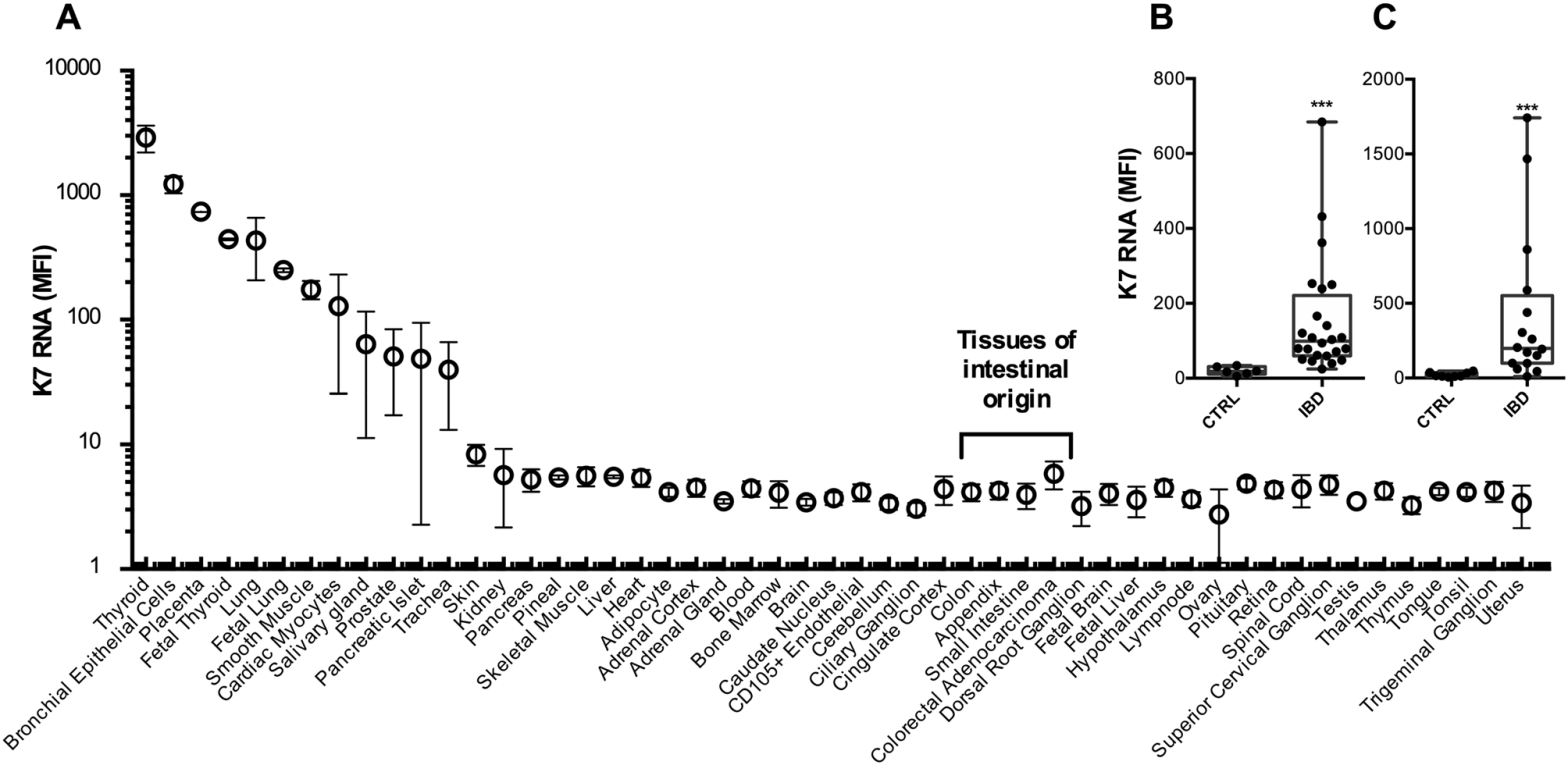
K7 mRNA is expressed in inflamed colon but not in healthy colon. (**A**) According to the tissue-specific mRNA expression in bulk RNAseq, dataset published by Su et al. 2004, K7 mRNA (probe 209016_s_at) is not found at detectable levels in samples of intestinal origin. (**B**, **C**) K7 mRNA is significantly upregulated in the colon of IBD patients. Data was downloaded from publicly available transcriptomes (**B** Arijs et al. 2009, E-GEOD-14580; **C**, Galamb et al. 2008, E-GEOD-4183) which include in total samples from both IBD patients (N = 40) and healthy controls (N = 14), where one dot represents a single individual. Boxes extend from 25 to 75th percent, line inside shows median value and whiskers min and max values. Statistical significance was measured by (**B**, **C**) Mann–Whitney test. *P < 0.05; ***P < 0.001.

To study the K7 mRNA expression, we analyzed the publicly available human gene atlas data^21^, which confirms that K7 mRNA is not significantly expressed in healthy colon, appendix or small intestine (Fig. 3A). To learn whether K7 expression in IBD is regulated at transcription or post translationally, we quantified the K7 mRNA data from two previous studies, stored in accessible bulk RNAseq data E-GEOD-14580 and E-GEOD-4183 in the ArrayExpress repository. One set include UC patients^22^ (Fig. 3B) and the other set include CD and UC patients^23^ (Fig. 3C). In both IBD datasets K7 mRNA expression was significantly increased in the colon of IBD patients (Fig. 3B,C). K7 is therefore increased both at protein and RNA level in IBD, indicating transcriptional level K7 regulation.

### The colonic K7 increase in IBD patients is associated with epithelial changes including erosion and ulcers

**Figure 4:**
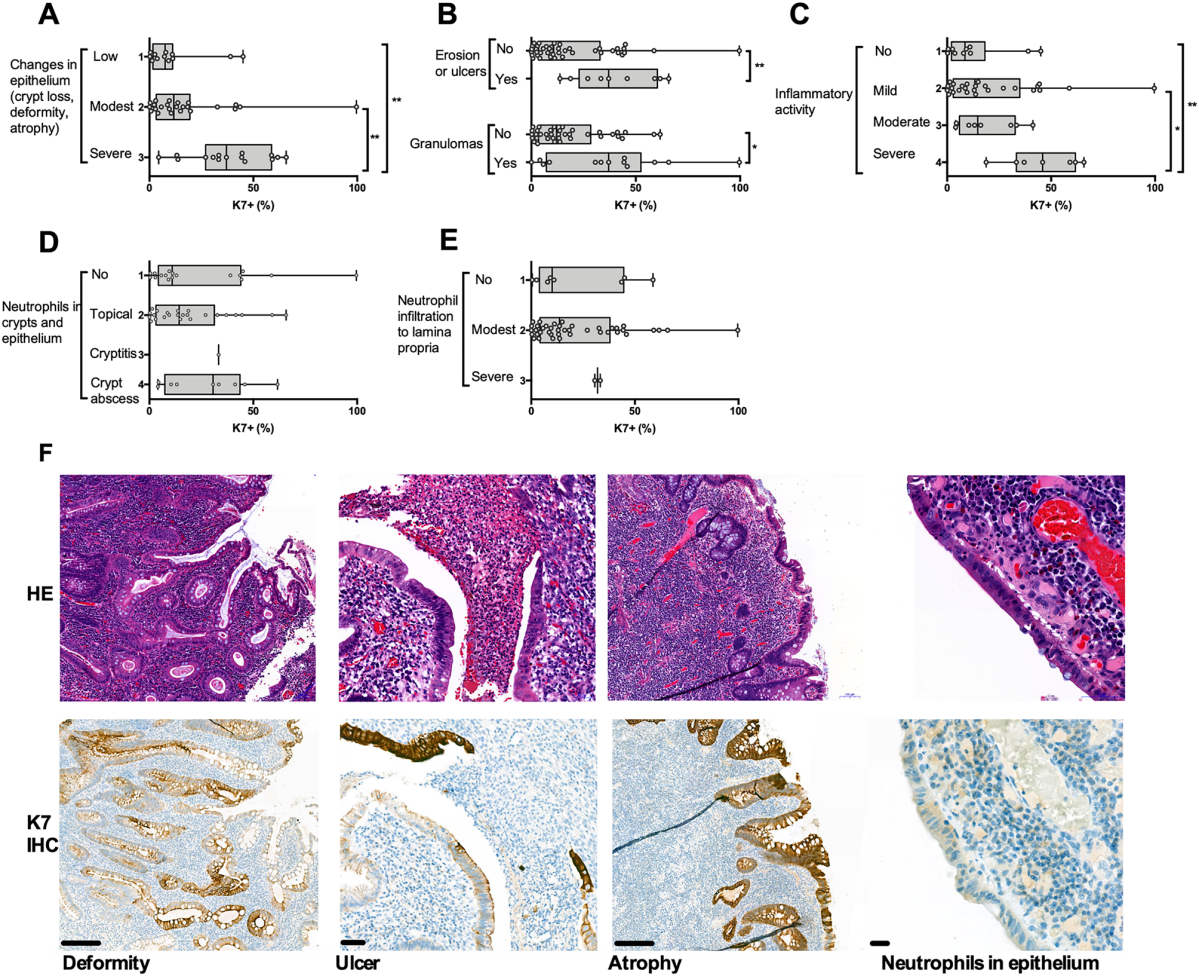
Severe pathological changes in epithelium induce colonic K7. Pathological changes in IBD sample annotations (aROI) in which K7 was quantified (two per patient) show that (**A**) major changes in epithelium, including atrophy, deformity, and crypt loss (**B**) as well as ulcers, erosion and granulomas, are associated with high K7 + percentage. (**C**) Increased inflammatory activity excluding ulcers did not change the percentage of K7 expressing cells, neither did the number of neutrophils in (**D**) epithelial cell layer nor in the (**E**) lamina propria. IBD patient samples presented here belong to cohort A. (**F**) Representative H&E and K7 IHC images of pathological changes are shown for deformity and atrophy (scale bar 200 μm), ulcer (50 μm), neutrophils in epithelium (20 μm). Boxes extend from 25 to 75th percent, line inside shows median values, whiskers min and max values, and grey dots individual sample annotations. The significance between more than two groups was measured using Dunn’s multiple comparison test. Mann–Whitney test in pairwise comparison. Correlations of two variables were estimated using linear regression analysis. *P < 0.05. **P < 0.01.

**Figure 5:**
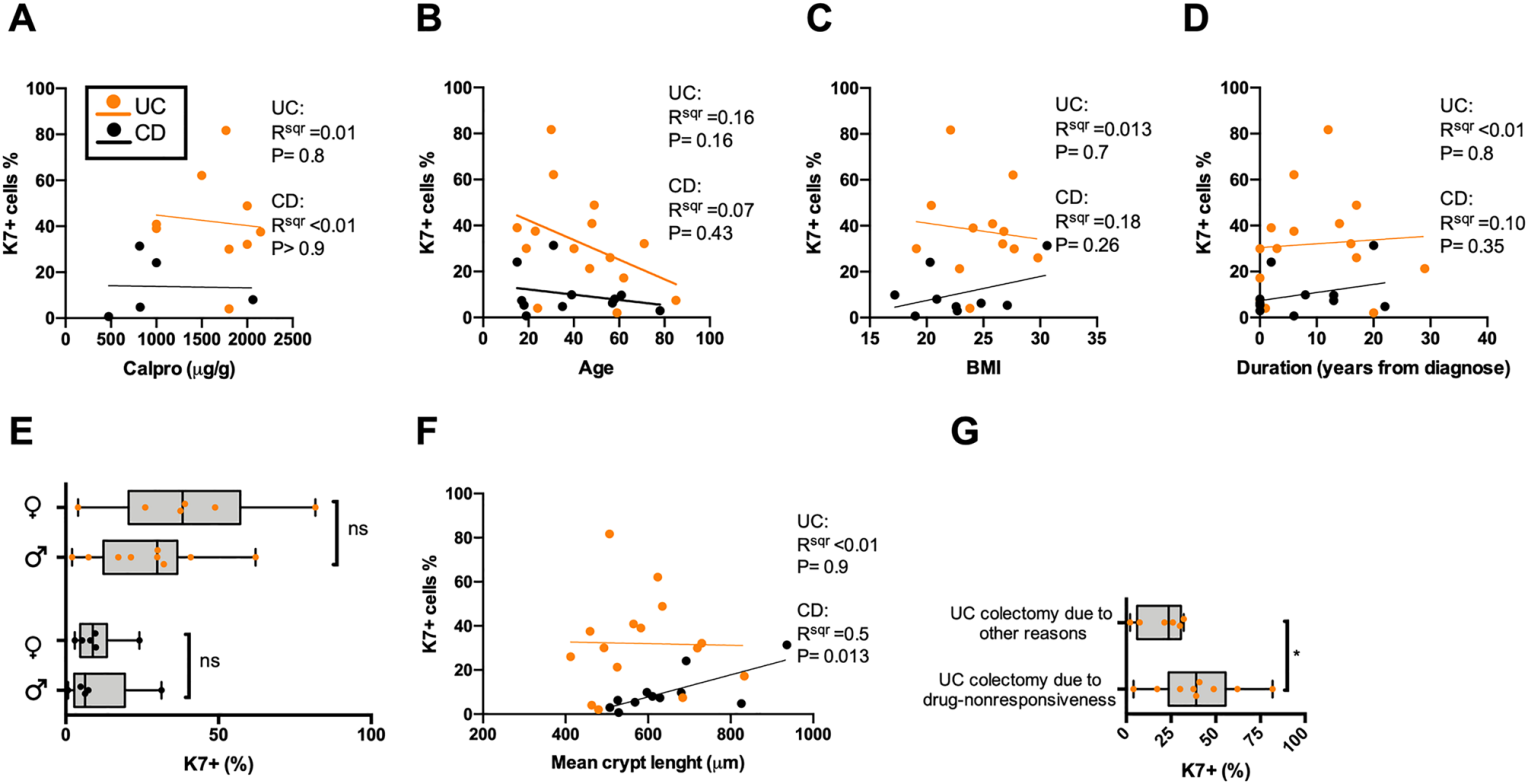
Correlation of clinical characteristics with K7 expression levels in IBD. (**A**) Fecal calprotectin (Calpro) levels prior to surgery had no correlation with colonic K7 levels, neither had (**B**) patient age, (**C**) BMI, (**D**) the duration of disease from onset to harvest nor (**E**) sex. (**F**) Mean top to bottom colon crypt length correlated with K7 in CD but not in UC. (**G**) K7 upregulation was the most pronounced in drug resistant UC patients. Orange dots in figures represent UC and black dots CD patients, illustrating the average percentage of K7+ cells in annotated areas. Samples presented here were derived from IBD patients in cohort A. Correlations of two variables were assessed using linear regression analysis. Mann–Whitney test was used in pairwise comparison. *P < 0.05.

To identify the conditions in which K7 is upregulated, the epithelial areas in which K7 levels were annotated and quantified (here called eROI), as well as the surrounding close proximity areas (aROI) of these epithelial annotations, were scored for their histopathological characteristics. The highest focal K7 + cell concentration was found in the samples with most severe epithelial changes and damage including crypt loss, atrophy, erosion and ulcers (all IBD samples in cohort A being pooled; Fig. 4A,B). In addition, samples with granulomas close to the epithelium had an increased number of K7 positive cells (Fig. 4B). In addition to epithelioid granulomas, microgranuloma lesions, also present in UC, were included^24^. Epithelial areas with high local inflammatory activity had more K7 positive cells than those with less activity (Fig. 4C). Other factors associated with the increased inflammation had no clear correlation with K7 (Fig. 4D–E). In spite of K7 levels being the highest in areas with severe disease manifestation and severe inflammatory activity, there was no correlation between K7 and the neutrophil numbers in the epithelium (Fig. 4D), or in the lamina propria (Fig. 4E). Representative areas with increased K7 close to ulcers, crypt atrophy and deformity are shown (Fig. 4F). In concordance with that the presence of neutrophils does not correlate with K7, stool calprotectin had no correlation to the K7 positivity in the epithelium in UC or CD (Fig. 5A). In addition, patient age, BMI, time from disease onset and sex did not correlate with the percentage of K7 positive cells (Fig. 5B–E).

**Figure 6:**
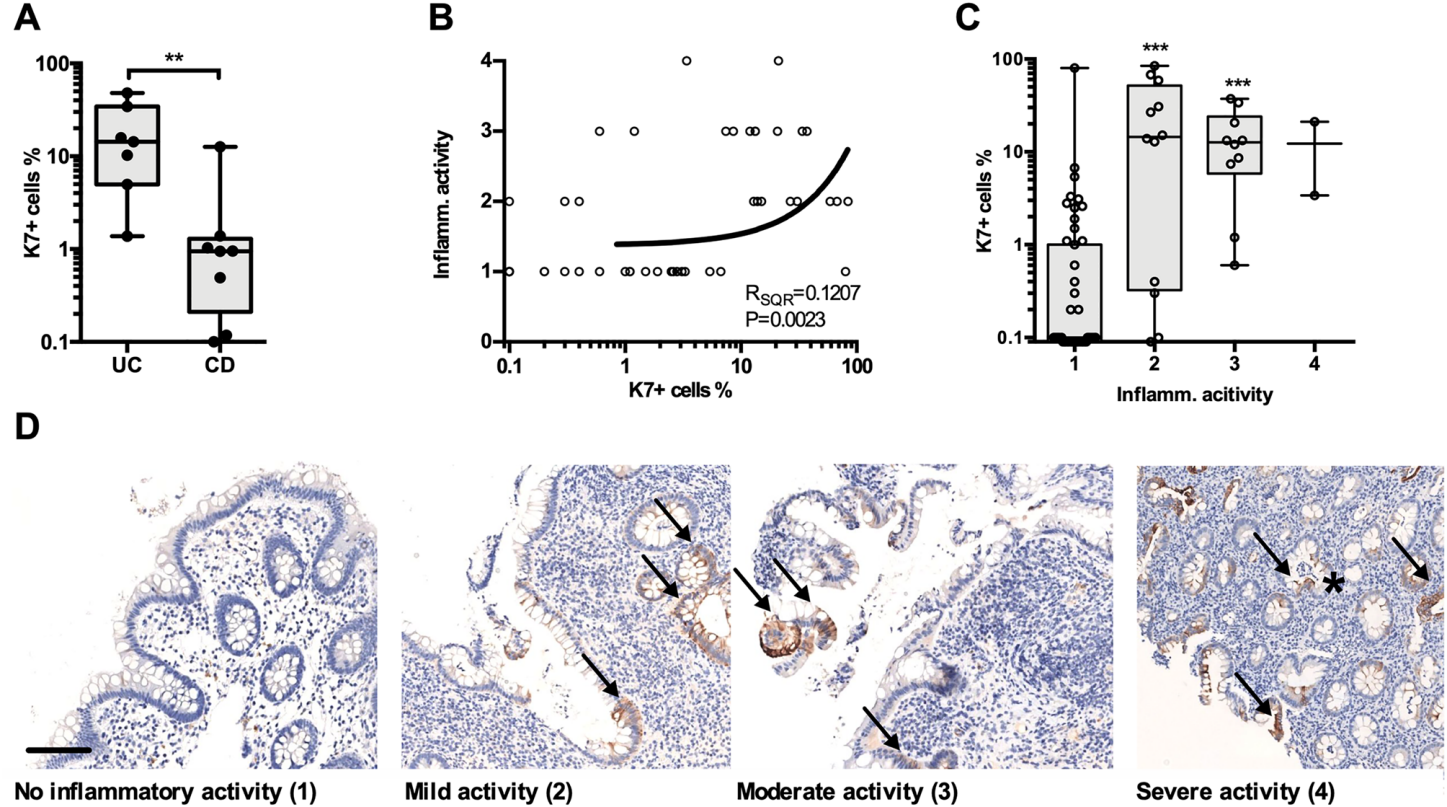
K7 is focally expressed in healthy epithelium of IBD patents, but the K7 positive cell percentage is yet lower than in inflamed areas. Samples were derived from UC (N = 7), and CD (N = 8) patients in cohort B, including total 75 biopsies. (**A**) The mean percentage of K7 positive cells was higher in UC than CD patients with drug responsive IBD. Each dot represents an average K7 + cell percentage among the biopsies collected from a single patient. (**B**) Increased local inflammatory activity correlated with the percentage of K7 + cell in colon biopsies. (**C**) The percentage of K7 + cells according to the inflammatory activity where biopsies were graded on a scale from 1 to 4 (1: no activity, 2: mild. 3: moderate, 4: severe) is shown. Circles represented individual biopsy sample in (**B**) and (**C**). (**D**) Representative K7 IHC staining (brown) in each inflammatory activity category is shown by arrows, and crypt abscess by asterisk. Correlation of two variables was assessed using linear regression analysis. Mann–Whitney test was used in pair way comparison. The significance between more than two groups was measured using Dunn’s multiple comparison test. **P < 0.01; ***P < 0.001.

Crypt length correlated positively with the number of K7 + cells in CD, but not in UC samples (Fig. 5F). Interestingly, the K7 + cell distribution in crypts did not have a clear location pattern, and while K7 + cells were often found in clusters next to each other (Fig. 2), sporadic cells with K7 positivity were also commonly found. Nevertheless, single K7 positive cells were also present in several microscopic colitis and control samples, but their total share of epithelial cells was below 1% (Fig. 1D). It is noteworthy that LC, CC and controls did not show any of the studied pathological changes in the epithelium, and crypt lengths were not increased. We were not able to associate the IBD-related K7 positivity to specific cell subtypes or regions in the colonic epithelium. For example, the obvious and abundant colonic goblet cells were found to be either K7 positive or K7 negative in the same patient and area (see e.g., Figs. 2, 6D and Supplementary Fig. 2). These observations and further cell type identification will require molecular level confirmation. Taken together, these findings suggest that K7 levels increase with epithelial damage.

When the UC patients were stratified further according to the drug resistant or non-resistant forms of the disease, patients whose colectomy was carried out due to drug resistance had more K7 positive cells compared to those whose colectomy was due to other reasons including cancer, severe dysplasia, and severe infection (Fig. 5G). Thus, the high percentage of K7 + cells in the colon epithelium could be associated with drug-resistant colitis. Considering the effect of specific drug treatments, we did not find significant difference in colonic K7 expression between drug receivers and non-receivers except for an increased K7 in cohort A CD patients receiving azathioprine (Table 1, Supplementary Fig. 1).

**Table 1:** Clinical characteristics and medication of patients.

Group	Number	Sex F/M	Age at harvest (median)	Years from onset to harvest (median)	BMI (median)	# of drug receivers: mesalazine	Corticosteroids	TNFα blockers	Azathioprine	Sulfasalazine	Methotrexate	Other
**Cohort A**												
Control	12	6/6	34–80 (57)	–	18.3–28.7 (27.6)*	1	0	0	0	0	0	
UC	15	6/9	15–85 (47)	0–29 (6)	19.1–29.8 (24.9)	12	14	5	6	2	0	^¶^
CD	11	6/5	17–78 (35)	0–22 (6)	17.2–38.1 (22.6)	5	5	3	3	1	2	
CC	8	6/2	40–85 (78)	0–9 (0)	23.4–23.5 (23.5)**	2	3	0	0	1	0	^¶¶^
LC	10	5/5	63–88 (78)	0–4 (0)	19.3–28.9 (25.3)	0	0	0	0	0	0	
**Cohort B**												
UC	7	6/1	19–39 (28)	0–14 (1)	17.5–24.5 (22.6)^§^	5	7	0	4	0	0	
CD	8	5/3	20–85 (34)	0–12 0)	19.5–32.1 (21.9)	1	8	0	3	0	0	^§§^

The numbers in the medical treatment columns indicate the number of patients receiving this medication within 1 month prior to tissue or biopsy harvest.

*Only 4 values available.

**Only 2 values available.

^¶^Single users of allupurinol, ursodiol and anabolic steroids.

^¶¶^Single users of leflunomide and hydroxychloroquine.

^§^Only 5 values available.

^§§^Single user of mercaptopurine.

### K7 increase is pronounced in inflamed parts of colon

To confirm whether the severity of IBD is pivotal for colonic K7 expression, we studied K7 levels in samples collected in a prospective study cohort (referred here as clinical cohort B) of patients with drug responsive IBD. Patients with UC had significantly higher percentage of K7 + epithelial cells compared to CD (Fig. 6A). When single biopsies were studied, a positive correlation between local inflammatory activity and K7 + fraction was found (Fig. 6B). This correlation, nevertheless, showed reasonable variation as R squared is only 0.12 which may be the reason that a significant difference in K7 expression was only seen between inflamed and non-inflamed samples when samples were stratified according to the inflammatory activity (Fig. 6B–D). Thus, no difference in K7 expression between low and severe local inflammation was found. (Fig. 6C–D). Several IBD patient samples lacking local inflammatory activity or other disease manifestation still had more K7 + cell than any of the healthy controls in cohort A (Figs. 1E, 6C) indicating that IBD may induce focal K7 expression in healthier parts of colon.

When cohort B (which did not include healthy control patients) results were compared to controls from cohort A, both UC and CD patients had significantly increased percentage of K7 positive epithelial cells (18.4% for UC and 2.2% for CD vs 0.27% for control, Figs. 1E and 6A). These values were generally lower than mean values measured in cohort A for colectomy patients (32.2% for UC and 9.2% for CD, Fig. 1E). Together these results indicate that the percentage of K7 + colonic cells is linked with the severity of IBD.

## Discussion

In this study we show in two separate patient cohorts that significant K7 expression in the colonic crypt epithelium is associated with the two main IBD diseases, UC and CD. The number of K7 + colonocytes is highest in close proximity to severe pathological changes in the colon and in drug-resistant UC, while there is no K7 expression in healthy controls or in microscopic colitis patient tissues. The lack of K7 expression in the normal colonic mucosa is supported by previous findings^1,25^ and thus, it has no known role in the healthy human colon. The K7 mRNA levels also increase in the colon in IBD patients compared to healthy colon, confirming transcriptional activation of K7 in IBD. In addition to healthy tissue, the majority of sporadic CRC are K7 negative and K7 found in metastasis is often used as a rationale against colorectal origin^26,27^. Recent studies suggested that K7 might indicate poor prognosis in some sporadic colorectal carcinomas^28^, and similarly in cervical adenocarcinoma^29^. Interestingly, K7 was found to be expressed as high as 45–70% of colitis-induced CRC^6,7^. This finding together with our results, suggests an epithelial switch inducing K7 expression in IBD, which then remains active in inflammation-induced colorectal carcinogenesis.

Although there was a slight correlation between total inflammatory activity and local K7 + cell percentage, it is unlikely that T cells or neutrophils would directly induce K7 as the number of neutrophils in the epithelium and lamina propria, and as stool calprotectin levels did not correlate with K7 + cells in UC and CD. Supporting this conclusion is that in LC, characterized by excess of lymphocytes within the epithelium, K7 was not increased over levels in healthy controls. CRC tissues are also accompanied by various immune cells^30^ and still K7 is not frequently detected in CRC tumors except in colitis-related cancers^31^. Therefore, it is also unlikely that an upregulated proliferative signaling would induce K7, supported by the lack of correlation between crypt elongation and K7 in UC samples, even if a mild correlation was seen in CD. Age, sex and BMI of patients did not affect the expression of colon K7, suggesting that sex steroids and white adipose tissue hormones are not among the most prominent inducers. Interestingly, we found that many IBD samples with particularly high K7 expression have histiocyte formed granulomas close to epithelium. Not much is known about the role of histiocytes affecting simple epithelial cell in keratin synthesis, and this result warrants further studies.

Keratins and other intermediate filaments are cytoprotective proteins and reportedly upregulated in various stress and regenerative conditions, which may explain the here described K7 neo-expression in the colonic epithelium of IBD patients^8,32^. Simple epithelial keratins including K7 have been shown to be upregulated or neo-expressed in patients and in mouse disease models, for example in kidney epithelial cells during renal injury^33^, in β-cells after diabetes induction^34^, and in hepatocytes of patients with cholestatic diseases^35^. Similarly, in mouse models of colonic stress and inflammation, simple epithelial keratins were upregulated, including K7 which was increased in a model of chronic colitis and in aging mice^19^. In cancers, simple epithelial keratin overexpression in general is associated with poor prognosis^36,37^. As IBD is a risk factor for CRC, the possible link between the colonic K7 expression in IBD and colitis induced carcinogenesis will require further analysis. In the embryonic tissue K7 is ubiquitously expressed^38^, but its expression is lost postnatally in several tissues, for example in differentiated human acinar cells of the pancreas^39^. It was suggested that K7 in the gut could be a cellular dedifferentiation marker^40^, supported by findings that it is expressed in fetal stomach^41^, but the exact biological role of K7 in the gut is not characterized, neither is its distribution between colonocyte subpopulations. In addition, the regulatory mechanism of epithelial keratins, including the induction of K7, is not known in detail to our knowledge.

IBD biomarkers are an intensively studied topic, but there is yet no reliable analysis to predict disease outcome^42^. Therefore, it was intriguing that high K7 levels were found in UC patients with poor response for drug treatments. Neutrophil produced stool calprotectin is currently a widely used biomarker for IBD^43^. However, K7 is produced in epithelial cells and thus may reflect the epithelial changes better than short term neutrophil activity. Here we show that in microscopic colitis in which elevated calprotectin concentrations are often found^44^ does not promote colonic K7. In the future, it will be important to study intestinal K7 expression in other inflammatory and infectious diseases to confirm its IBD specificity. While the correlation between fecal calprotectin and epithelial K7 was not found in the present study, their combinatory diagnostic power needs to be studied further in a larger cohort, especially regarding the drug responses and disease-prediction capacity. The utilization of more than one biomarker for IBD is commonly considered a benefit for diagnosis^45^ and K7 is a potential IBD biomarker that also distinguishes IBD from microscopic colitis. Many pathology laboratories are already well-prepared to carry out K7 immunohistochemistry^46^. In addition, previous studies have shown that fecal assays might be a feasible method to monitor keratin expression in the colonic epithelium although data from K7 is still lacking^47,48^. Therefore, a study to simultaneously measure fecal K7 and calprotectin would be of high interest to evaluate the utility of K7 as a colon inflammatory disease biomarker.

In conclusion, the results presented here indicate K7 as a novel and specific marker of IBD in the colon, and show that the increase of K7 was the most pronounced in the proximity of epithelial damage and in patients with poor drug-response. K7 is a promising potential IBD diagnostic marker candidate as it is not expressed in a healthy intestinal tissue and its upregulation is measurable both on protein and mRNA level.

## Materials and methods

### Patient material

Cohort A FFPE colon samples were obtained from the Auria Biobank (Turku, Finland). UC and CD samples were harvested during colectomy or ileal resection from areas between sigmoid and ascending colon. CC, LC and control biopsies were harvested during ileocolonoscopy. The exclusion criteria for control tissues were inflammatory and neoplastic intestinal diseases. Transport, handling, and storage of the paraffin-embedded tissue samples were carried out according to the biobank standard procedures. The medical history of each patient, relevant to IBD, was filed and information was stored in encoded format and kept anonymous. The research project was authorized by the Auria Biobank’s Scientific Steering Committee (AB17-6901) and Hospital District of Southwestern Finland (decision T05/032/19). Study was carried out in accordance with ethical guidelines of Åbo Akademi University and Turku University Hospital. Bulk RNAseq data (GEOD-14580 and E-GEOD-4183) was accessed using the biogps.org gene annotation portal^49^.

Patient cohort B samples consisting of mucosal biopsies taken during ileocolonoscopy were acquired from a prospective study (Clinicaltrials.gov identifier NCT02364973) including patients in Southwestern Finland with diagnosed or suspected IBD. The protocol was approved by the ethics committee of the Hospital District of Southwest Finland (decision 66/1801/2014; §264), and the research grant of Turku University Hospital was received by the director of the Division of Medicine (decision TO5/039/14; 13604). Patient recruitment, exclusion criteria, diagnostic methods and sample collection have been presented elsewhere^50^. All patients were informed about the nature of the study, and they signed an informed consent before participating in the study. Clinical characteristics of cohort A and B are described in Table 1.

### Immunohistochemistry

The tissue samples were fixed in 4% phosphate-buffered formaldehyde and embedded in paraffin according to standard procedures. K7 immunohistochemistry (IHC) staining was carried out from 5 µm rehydrated sections with an antibody to K7 (clone SP52, Roche Diagnostics, Rotkreuz, Switzerland). The protein visualization was carried out using anti-mouse secondary antibody and 3,3′-Diaminobenzidine (DAB) as a chromogen and hematoxylin counter-stain. Calprotectin immunoassays were carried out from stool samples in Turku University Hospital, Laboratory Division, Clinical Chemistry.

### Digital image analysis

The slides were scanned to digital images (Pannoramic 1000, 3D HISTECH, Budapest, Hungary) and the mean intensity of cellular K7, followed by quantification of K7 positive cells were measured using the QuPath 0.2.3 bioimage analysis application^51^. The epithelial cell layer region of interest (eROI, at least two distinct areas per samples) was selected manually. eROIs including full crypts were prioritized when available (Supplementary Fig. 2). Each eROI area contained at least 1000 cells per single biopsy and over 2000 cells in total per patient were annotated and identified by QuPath cell detection tool. K7 expression analysis was based on the mean intensity of cellular DAB staining. In addition, every cell in eROI areas was ranked according to the K7 intensity and given a value from 0 to 3, where 0: no K7 present, 1: low K7, 2: medium and 3: high K7 present, using the QuPath positive cell detection (DAB cell mean OD) tool. The lowest K7 threshold was based on barely visible cytoplasmic DAB staining (Fig. 1D). The cells ranked from low to high K7 are referred here as K7 positive (K7+). The mean top to bottom crypt length was measured as an average of at least four crypts per sample.

The K7+ percentage was quantified from each eROI and the surrounding close proximity area (aROI, Supplementary Fig. 2) was used to characterize pathological changes. For grading the severity of IBD, histological analysis were proceeded according to ﻿The European Crohn's and Colitis Organization guidelines^52^, also approved by the ethics committee of the Hospital District of Southwest Finland (decision 66/1801/2014; §264). The histopathological analysis was carried out in from parallel H&E-stained samples of each aROI by an analyzer not aware of the K7 expression in that area. Inflammatory activity score summarized both the total amount of granulocytes and their activity which were graded into four classes: no increase, mild, moderate (cryptitis) and severe (crypt abscesses, Fig. 6D). Neutrophil infiltration in the epithelium was graded into four classes: none, topical neutrophils, cryptitis and crypt abscesses. Neutrophil presence in lamina propria was graded into three classes: none, modest and severe infiltration. The presence of ulcers, erosion, or granulomas (including microscopic granulomas) was assessed in a similar manner in the aROI (Supplementary Fig. 2). Epithelial changes including crypt deformation, atrophy and crypt loss were graded to three classes: low, modest, and severe.

### Statistical analysis

The difference between more than two groups was measured using Kruskal–Wallis test, followed by Dunn’s test. The difference between two factors was evaluated using Mann–Whitney test. The correlation of two factors was studied using linear regression analysis. All statistical analyses were performed using GraphPad Prism (GraphPad Software Inc., San Diego CA, USA).

## Supplementary Information

Supplementary Figures.

## Data Availability

The datasets generated during the current study are available in the etsin.fairdata.fi repository (https://doi.org/10.23729/edd4abd6-9d04-491c-b11a-46aff225ce45). A few clinical, patient sensitive parameters on research data are not made publicly available due to privacy of patients but are available from authors by permission of Auria Biobank and Hospital District of Southwest Finland on reasonable request. RNA datasets analyzed here are available at biogps.org (E-GEOD-14580 and E-GEOD-4183 in the ArrayExpress repository).
