# Supplementary material for: Colonocyte keratin 7 is expressed de novo in inflammatory bowel diseases and associated with pathological changes and drug-resistance
Source: Sci Rep. 2022 Dec 23;12:22213. doi: 10.1038/s41598-022-26603-2 (PMC9789078; doi:10.1038/s41598-022-26603-2)
Supplement: Supplementary file 1 — Supplementary Figures. [file 41598_2022_26603_MOESM1_ESM.docx]

**Supplementary figure 1**


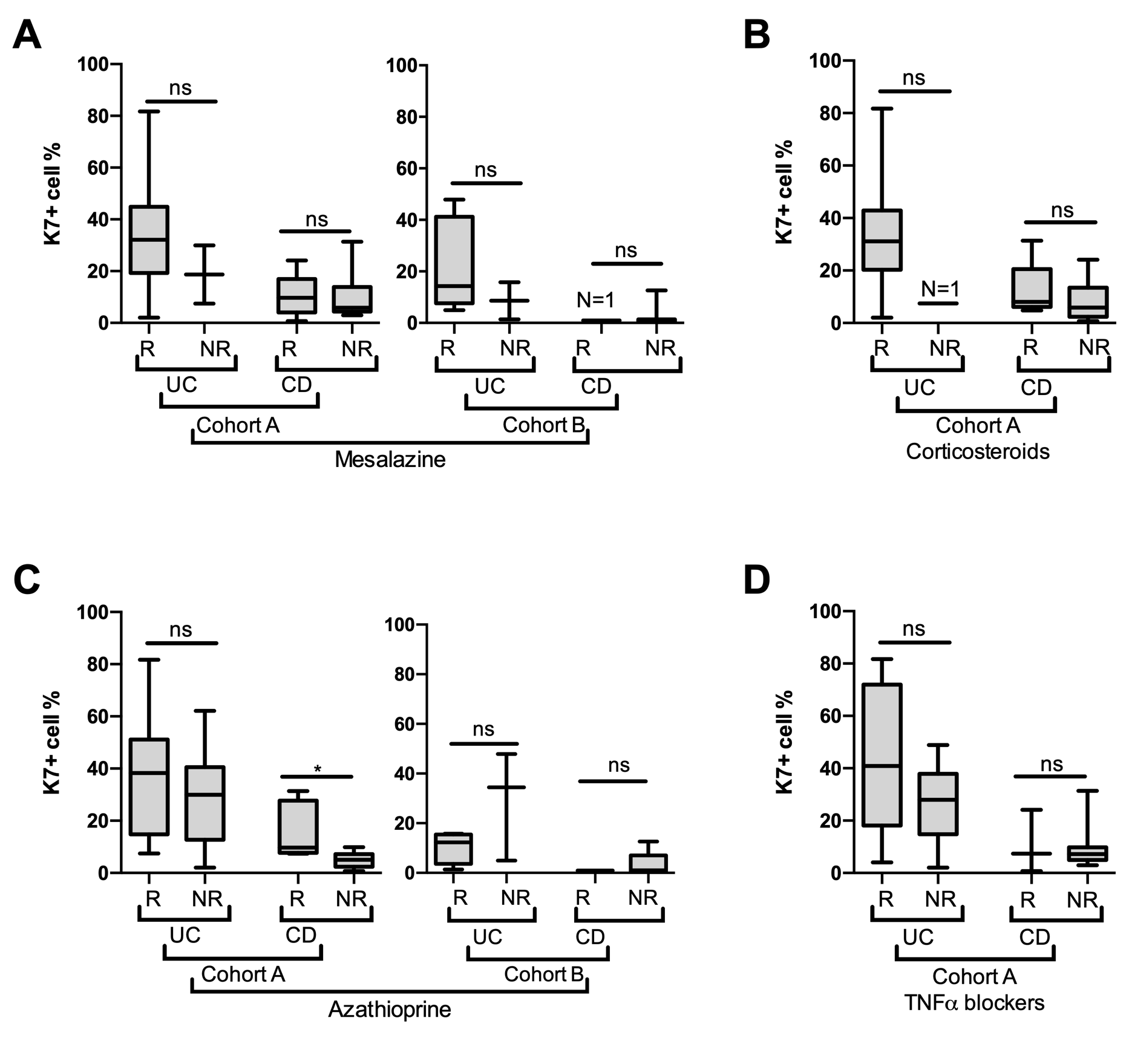


**Supplementary figure 1 legend: The effect of received medication on colonic K7 in IBD.**  The mean colonic K7+ cell percentage in ulcerative colitis (UC) and Crohn’s disease (CD) patients of both cohorts A and B are stratified according to the IBD drugs (A) mesalazine, (B) corticosteroids, (C) azathioprine, and (D) TNFα blocker, received 1-month prior to sample collection (for N see Table 1). In cohort B, all patients received corticosteroids and none of them TNFα blockers. R=drug receiver, NR=non-drug receiver. Boxes extend from 25th to 75th percent, line inside shows median values and whiskers min and max values. The significance between receivers and non-receivers was carried out using Mann-Whitney test. ﻿*P < 0.05.

**Supplementary figure 2**


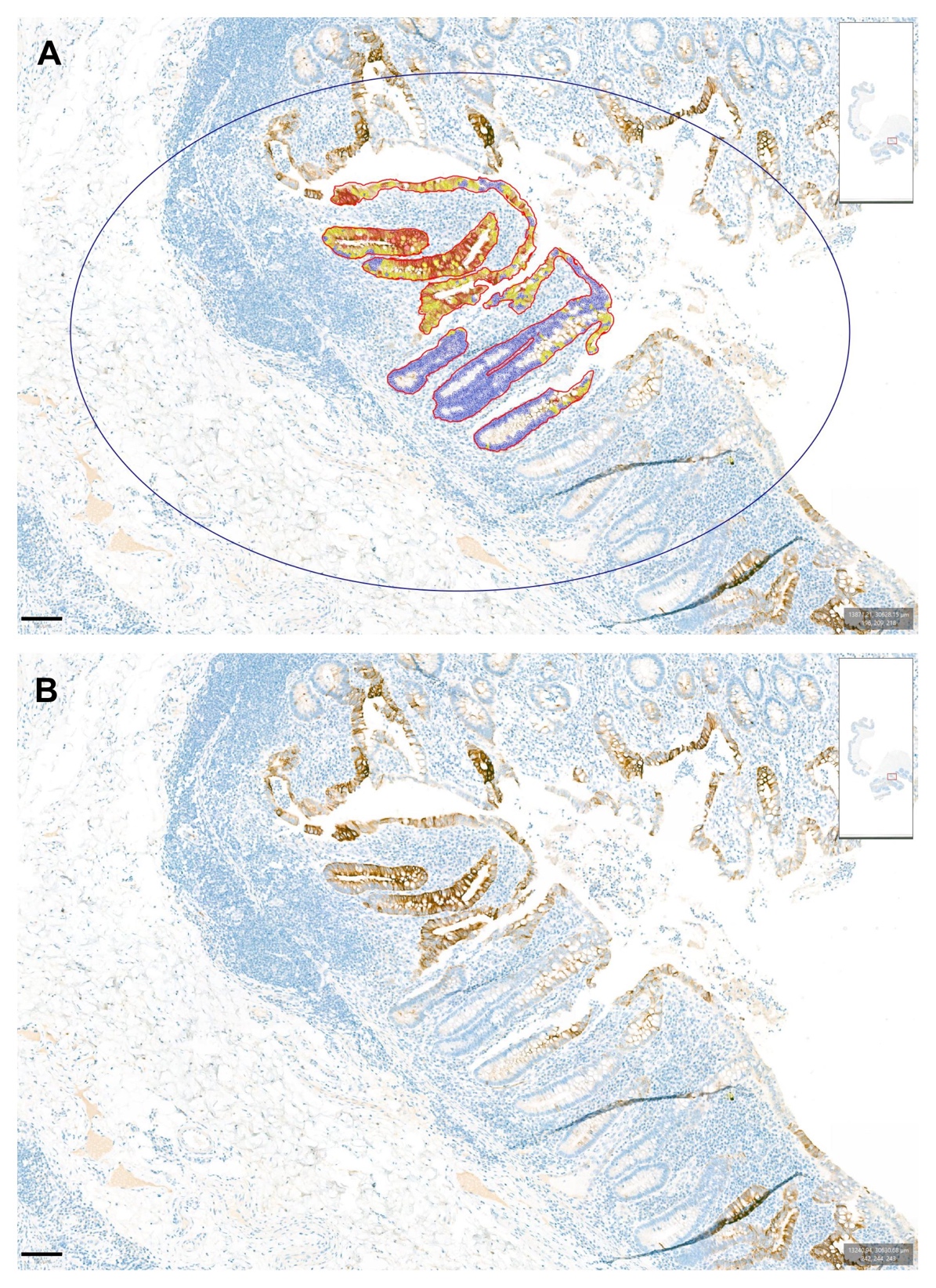


**Supplementary Figure 2. Representative example of colonic K7 staining, ROIs and epithelial annotations in an IBD patient tissue section**. (A) The manually annotated, red-lined area (eROI) consists only of epithelial cells. The epithelial cells are recognized and cellular K7 expression from immunohistochemically stained (brown color) and scanned patient colon sections is identified and quantified by QuPath 0.2.3 software: blue cells are K7 negative, while yellow (K7 low), orange (K7 medium), and red (K7 high) cells are considered K7 positive. The large blue circle marks the ROI area around annotations (aROI), in which the pathological scoring was carried out. (B) Figure B shows only the K7 staining seen in A without eROI and aROI annotations. Scale bar = 100 μm.
